# Graves’ hyperthyroidism accompanied with acute hepatitis B virus infection: an extrahepatic manifestation?

**DOI:** 10.1186/s12985-016-0537-z

**Published:** 2016-05-20

**Authors:** Wei Cui, Baocheng Deng, Wen Wang, Pei Liu

**Affiliations:** Department of Infectious Diseases, the First Affiliated Hospital, China Medical University, Shenyang, China

**Keywords:** Graves’ hyperthyroidism, HBV infection, Extrahepatic manifestation, Antiviral

## Abstract

**Background:**

Although hepatitis B virus (HBV) primarily affects hepatocytes, it has also been shown to cause complications in the skin, joints, muscles, and kidneys. Thyroid dysfunction is uncommon in cases of acute HBV infection.

**Case presentation:**

In this report, we describe a case of a 46-year-old woman with incipient acute hepatitis B virus (HBV) infection who presented clinically with Graves’ hyperthyroidism. She showed typical symptoms of hyperthyroidism, and laboratory tests revealed high levels of HBV DNA and alanine transaminase (ALT). The patient was not administered with antithyroid medicine or radioiodine, but she was given antiviral therapy and symptomatic treatment with propranolol. Follow-up studies showed that as the HBV DNA levels decreased, the thyroid function recovered.

**Conclusion:**

Graves’ disease maybe an extrahepatic manifestation of acute HBV infection. Antiviral therapy is likely to be beneficial for this condition as without severe thyrotoxicosis.

## Background

Hepatitis B virus (HBV) infection is particularly prevalent in China. Although HBV primarily affects hepatocytes, it has also been shown to cause complications in the skin, joints, muscles, and kidneys [1]. The extrahepatic manifestations are nonspecific for HBV infection, and the associated symptoms are mainly based on immune complex reactions that occur in other organs.

Graves’ disease is the most common cause of hyperthyroidism. It is characterized by the presence of autoantibodies against various thyroid components, as well as by an inflammatory cellular infiltrate of variable severity within the gland. Although the etiology and pathogenesis of Graves’ hyperthyroidism are still not clear, environmental factors including viral infection are suggested to trigger or to be involved in its manifestation [2, 3].

Although patients with both Basedow-Graves’ hyperthyroidism and viral hepatitis have also been observed, concurrent thyroid dysfunction is uncommon in cases of acute viral hepatitis. Awareness and recognition of this manifestation are important to facilitate early diagnosis and treatment.

## Case presentation

A 46-year-old female presented with recurrent and aggravated weakness, palpitation, and distending pain in the eyes for 10 days in April 2009. The patient experienced fatigue and palpitation 1 month previously. She experienced weak legs and shaking hands, and was examined twice in a local clinic. The plasma potassium level was 2.57–3.0 mM, which is less than normal physiological levels (3.5–5.5 mM). The patient was administered with a potassium supplement, and the above symptoms disappeared. Ten days before admission (April, 2009), the patient again showed weakness, palpitation, shaking hands, distending pain in the eyes, and no appetite. Diagnosis at the Endocrine Department of the clinic showed abnormal thyroid and liver functions as well as a high level of fasting blood glucose (FBG). The patient was then hospitalized for systemic examination. Inspection of her disease course revealed that after the occurrence of the above symptoms, she had polydipsia, no fever, no hyperhidrosis, no dysphoria, and no increasing food intake or rapid hungering. The patient lost 5 kg in the 15 days before admission.

The patient had no history of viral hepatitis (HBV markers was negative 3 years ago), tuberculosis, high blood pressure, diabetes mellitus, or thyroid disease. In addition, there was no history of surgery, trauma, blood transfusion, or HBV carriers among her family members. Physical examination showed conscious admission, a pulse rate of 98 beats per min, and bright eyes. The exophthalmometric values of her left and right eyes were both 18 mm. There was no Stellwag’s sign, no von Graefe’s sign, no Joffroy’s sign, and no Mobiud sign. The thyroid gland was pliable and had mild swelling. There was no thyroid nodule or tenderness. Vascular bruit was negative, and there were no liver palms or spider angioma. In addition, there were no abnormalities of the heart, lung, or abdomen, and there was no swelling of the legs. Laboratory examination showed the following (Table 1): HbsAg^+^; HBeAg^+^; HBcAg IgM^+^; HBV DNA >5.0 × 10^7^ copies/mL; an alanine transaminase (ALT) level of 351 U/L; negative for HAV-Ab, HCV-Ab, HEV-Ab,EBV-Ab,CMV-Ab and CMV DNA; increased levels of free triiodothyronine (FT3, 18.13 pM) and free thyroxine (FT4, 39.98 pM); decreased levels of thyroid-stimulating hormone (TSH) (0.0364 mIU/L), TRAb^+^, and TPO Ab^-^; and an abnormal oral glucose tolerance test. The color Doppler flow imaging (CDFI) of the thyroid showed a swollen thyroid gland and lymph nodes of the bilateral neck, while the CDFI of the liver was normal. The patient was diagnosed acute HBV infection and Graves’ hyperthyroidism.

**Table 1:** Laboratory test results on admission

Laboratory test values on admission	Value	Reference values
Biochemical tests of the liver		
ALT (U/L)	351	4–43
AST (U/L)	186	1–38
T-BIL (μM)	11.5	3.4–20.5
ALP (U/L)	136	35–100
γ-GT (U/L)	92	7–45
Thyroid Function		
FT3 (pM)	18.13	2.6300–5.7000
FT4 (pM)	39.98	9.0100–19.0500
TSH (mIU/L)	0.0364	0.3500–4.9400
TRAb	11.23	<1.0
Virological aspects of HBV		
HBsAg	+	-
HBsAb	-	-
HBeAg	+	-
HBeAb	-	-
HBcAb-IgM	+	-
HBV DNA (copies/mL)	>5.0 × 10^7^	<5.0 × 10^2^
Oral glucose tolerance test		
Glu (0, 30, 60, 120, 180 min) (mmol)	5.97, 14.73, 16.12, 15.85, 11.72	3.80–5.80
Insulin (0, 30, 60, 120, 180 min) (mIU/L)	11.47, 78.04, 96.75, 96.95, 46.85	2.60–11.10
C-Peptide (0, 30, 60, 120, 180 min) (pM)	658.89, 2490.57, 3299.40, 3842.60, 2926.13	260.00–629.00

As a middle-aged female who was TRAb positive, the patient belongs to the population with a high incidence of autoimmune diseases. In addition, because she had no history of hyperthyroidism or HBV, and both appeared at once, the incipient hyperthyroidism was considered as an extrahepatic manifestation of acute HBV infection. Therefore, the patient was not given antithyroid medicine or radioactive ^131^I therapy but only propranolol (10 mg, tid, po), as a symptomatic treatment, and entecavir (0.5 mg per day, os) to treat the HBV infection. The thyroid and liver functions as well as HBsAg levels were monitored. After 12 weeks of treatment, the FT3 level decreased to 3.80 pM, the FT4 level decreased to 12.23 pM, the TSH level increased to 1.4899 mIU/L, the HBV DNA level decreased to 8100 copies/mL, the ALT level was 112 U/L, the FBG level was 5.35 mM, and the postprandial blood glucose (PBG) level was 7.19 mM. At 24 weeks post-treatment, the HBV DNA level became undetectable, the HBsAg and HBeAg test results were negative, while the detection of HBeAb became positive, and that of HBsAb remained negative. In addition, the ALT level was 16 U/L, and the FBG, PBG, and thyroid functional parameters ranged within normal levels. Therefore, antiviral entacavir was stopped and the patient continued to be monitored. At 48 weeks post-treatment, HBV DNA remained undetectable, the ALT level stabilized at 17 U/L, HBsAg and HBeAg remained negative, and HBsAb became positive. In addition, the patient had normal thyroid functions. At the last follow up at 3 years post-treatment, the TSH, FBG, and ALT levels were normal and the HBsAg test was negative.

## Discussion

Herein, we report a case of Graves’ hyperthyroidism that has a high likelihood to be associated with acute HBV infection. As a hepadnavirus, HBV primarily affects hepatocytes, leading to a number of hepatic complications (fibrosis, cirrhosis, and hepatocellular carcinoma). Although uncommon, several immune-mediated extrahepatic manifestations may develop during both acute and chronic HBV infection [1, 4]. These extrahepatic manifestations are nonspecific for HBV infection, and several of them are now well established, including serum sickness-like syndrome, glomerulonephritis, polyarthritis, and dermatological conditions as well as vascular and neurological complications [5].

Graves’ disease is a unique immune system disorder characterized by cell proliferation and excess function that results in the overproduction of thyroid hormones (hyperthyroidism). Hyperthyroidism in Graves’ disease is caused by autoantibodies to the thyrotropin receptor (TRAbs), and measurement of these TRAbs can be useful for disease diagnosis and management. Because TRAbs are not controlled by the negative feedback system, stimulation of the thyroid often leads to the clinical thyrotoxic state of Graves’ disease. Although the production of TRAbs in Graves’ disease remains unclear, infections with various viruses, such as Coxsackie virus [6], hantaan virus [7], and Epstein-Barr virus (EBV) [3], have been implicated in the induction of Graves’ disease. However, to date, there are no reports regarding the direct association between Graves’ disease and HBV infection.

In the 1990s, extrahepatic HBV DNA sequences in patients with acute HBV infection have been identified by Southern blot hybridization analysis by Yoffe et al. [8]. HBV nucleic acids were demonstrated in the lymph nodes, spleen, gonads, thyroid gland, kidneys, pancreas, and adrenal glands, with the most intense signal of hybridization observed with DNA extracted from the lymph nodes. This extrahepatic distribution of viral DNA suggests that HBV infection may cause diseases of other organs. Moreover, the association of subacute thyroiditis with acute HBV infection has been reported [9]. In addition, direct evidence of the presence of viruses or their components in the organ are available for retroviruses (HFV) and mumps in subacute thyroiditis as well as for retroviruses (HTLV-1, HFV, HIV, and SV40) in Graves’ disease [10]. Several patients with both Basedow-Graves’ hyperthyroidism and viral hepatitis also have been observed by Mancini et al. [11], and it was hypothesized that this association could be explained by the individual genetic pattern in which an immunological fragility conditioned a predisposition to autoimmune diseases. These reports support the likelihood that HBV infection might cause autoimmune-related thyroid disease and that the association may not be coincidental.

The patient presented in this case had no history of hyperthyroidism or hepatitis B and no family history of HBV infection. The case exhibited typical symptoms of hyperthyroidism, like palpitation and shaking hands. Physical examination revealed exophthalmos, thyroid gland swelling, and no signs of chronic hepatitis. Laboratory examinations showed both Graves’ disease and HBV infection and HBsAg turned negative in 24w. These data support the diagnosis of Graves’ hyperthyroidism accompanied with acute HBV infection. Although there was a lack of direct evidence that this case of hyperthyroidism was induced by acute HBV infection because a thyroid biopsy was not taken, the recovery of thyroid function with the clearance of HBV DNA indicates that it had a high likelihood being an extrahepatic manifestation of acute HBV infection.

Treatment of Graves’ disease complications during acute HBV infection is a challenge for clinical physicians. Generally, most acute HBV infections (90 %) are self-limiting with clearance of the virus [12]; therefore, there is no need for antiviral treatment. However, antiviral therapy is required for patients with advanced liver disease, such as liver failure, or chronic HBV infection. To date, there is little information about whether antiviral treatment is needed for those with acute HBV infection and kidney or thyroid complications. It has been shown that antiviral therapy is beneficial for the recovery of proteinuria in HBV-associated glomerulonephritis [13]. In our case, the patient was diagnosed with acute infection with a high HBV DNA titer and the disproportionally low ALT level as compared with most acute infections (500–1000 U/L [14]) indicates a high tendency for the patient to develop chronic HBV infection. In addition, HBV replication plays an important role in the pathogenesis of HBV-related extrahepatic syndromes. As antiviral therapy is helpful to reduce the viral replication and improve the damage of extrahepatic disease [15–17], the patient was administered with entecavir. As for Graves’ disease, common treatments include antithyroid drugs (ATDs) (e.g., propylthiouracil and tapazole), radioiodine, and surgical resection. The selected treatment is based on the patient’s age, the extent of thyroid swelling, and whether the patient is pregnant or has exophthalmos or heart, liver, or kidney disease. ATDs are the first choice of treatment, while for patients with allergies to medication or a poor outcome, radioiodine should be used. In a previous report, therapeutic plasmapheresis and thyroidectomy were used to treat a 31-year-old man with preexisting hyperthyroidism and HBV infection due to worsened hepatic function and elevated thyroid hormone levels (thyrotoxicosis) [18]. As the woman in our case exhibited hyperthyroidism with abnormal liver function, we thought that the use of ATDs might aggravate liver injury. In addition, given that the patient had only mild swelling of thyroid and the thyrotoxicosis was not quite severe, the patient was not administered with ATDs but only propranolol as a symptomatic treatment together with antiviral entecavir. Follow-up examination revealed that this was a proper treatment because the thyroid function recovered as the HBV DNA level decreased.

## Conclusions

In conclusion, the recovery of thyroid function with the clearance of HBV DNA in our case implicates that autoimmune Graves’ disease is an extrahepatic manifestation of acute HBV infection. Antiviral therapy to HBV may be a rapid, reliable, and effective way to treat those patients who have mild thyroid diseases associated with acute HBV infection.
